# Oral cholera vaccine coverage during a preventive door-to-door mass vaccination campaign in Nampula, Mozambique

**DOI:** 10.1371/journal.pone.0198592

**Published:** 2018-10-03

**Authors:** Cynthia Semá Baltazar, Florentina Rafael, José Paulo M. Langa, Sergio Chicumbe, Philippe Cavailler, Bradford D. Gessner, Lorenzo Pezzoli, Américo Barata, Dores Zaina, Dortéia L. Inguane, Martin A. Mengel, Aline Munier

**Affiliations:** 1 Instituto Nacional de Saúde, Ministry of Health, Maputo, Mozambique; 2 Agence de Médecine Préventive, Abidjan, Côte d’Ivoire; 3 Agence de Médecine Préventive, Ferney-Voltaire, France; 4 Agence de Médecine Préventive, Paris, France; 5 World Health Organization, Geneva, Switzerland; 6 Direcção Provincial de Saúde, Ministry of Health, Nampula, Mozambique; 7 Direcção de Saúde de Cidade, Ministry of Health, Nampula, Mozambique; Public Health England, UNITED KINGDOM

## Abstract

**Background:**

In addition to improving water, sanitation and hygiene (WASH) measures and optimal case management, the introduction of Oral cholera vaccine (OCV) is a complementary strategy for cholera prevention and control for vulnerable population groups. In October 2016, the Mozambique Ministry of Health implemented a mass vaccination campaign using a two-dose regimen of the Shanchol™ OCV in six high-risk neighborhoods of Nampula city, in Northern Mozambique. Overall 193,403 people were targeted by the campaign, which used a door-to-door strategy. During campaign follow-up, a population survey was conducted to assess: (1) OCV coverage; (2) frequency of adverse events following immunization; (3) vaccine acceptability and (4) reasons for non-vaccination.

**Methodology/Principal findings:**

In the absence of a household listing and clear administrative neighborhood delimitations, we used geospatial technology to select households from satellite images and used the support of community leaders. One person per household was randomly selected for interview. In total, 636 individuals were enrolled in the survey. The overall vaccination coverage with at least one dose (including card and oral reporting) was 69.5% (95%CI: 51.2–88.2) and the two-dose coverage was 51.2% (95%CI: 37.9–64.3). The campaign was well accepted. Among the 185 non-vaccinated individuals, 83 (44.6%) did not take the vaccine because they were absent when the vaccination team visited their houses. Among the 451 vaccinated individuals, 47 (10%) reported minor and non-specific complaints, and 78 (17.3%) mentioned they did not receive any information before the campaign.

**Conclusions/Significance:**

In spite of overall coverage being slightly lower than expected, the use of a mobile door-to-door strategy remains a viable option even in densely-populated urban settings. Our results suggest that campaigns can be successfully implemented and well accepted in Mozambique in non-emergency contexts in order to prevent cholera outbreaks. These findings are encouraging and complement the previous Mozambican experience related to OCV.

## Introduction

Cholera is a neglected tropical disease, characterized by profuse watery diarrhea caused by infection of the intestine with the bacterium *Vibrio cholerae*. Approximately 1.3 billion people are at risk of cholera in endemic countries. An estimated 3 million cases and 95,000 deaths occur each year around the world [1], particularly in endemic countries where access to water and sanitation infrastructure is inadequate [2,3], including Mozambique [4,5]. The use of oral cholera vaccine (OCV) has become an essential strategy for prevention and control of cholera [6,7]. The World Health Organization (WHO), created in July 2013 a global OCV stockpilewas created to encourage vaccine use for underserved populations [8] and ensure rapid access to OCVs in outbreak situations, humanitarian emergencies, and endemic areas [5]. Three OCVs are prequalified by the WHO to be used: Dukoral (Valneva Sweden AB), Shanchol (Shantha Biotechnics Limited, India), and Euvichol (Eubiologics Co., Ltd., Korea) [8]. The immunization schedule consists of two doses given at an interval of at least two weeks.

The mechanism to allocate vaccines in emergency situations (i.e. outbreak response or humanitarian crisis) is managed by the International Coordinating Group (ICG), composed by Médecins Sans Frontières (MSF), the International Federation of Red Cross/Crescent (IFRC), Unicef, and the WHO; while the allocation of OCV in non-emergency (i.e. vaccination in cholera endemic “hotspots”) is coordinated by the Global Task Force on Cholera Control (GTFCC), a WHO coordinated network of partners active in controlling cholera [9]. According to the last WHO’s report, since creation of the stockpile, as of April 2017, almost 8 million doses of OCV have been shipped for the implementation of more than 40 OCV vaccination campaigns in 14 countries [10] including Cameroon, Democratic Republic of the Congo, Ethiopia, Guinea, Haïti, Iraq, Malawi, Niger, South Sudan and Zambia [11–16].

Mozambique is considered as endemic for cholera with recurrent outbreaks occurring almost every year. The outbreaks are more frequent in the northern part of the country, including in Nampula city [5]. Nampula is the capital city of Nampula province, the second most crowded province in the country, with approximately 2 million inhabitants. Nampula province gathers 21 districts including Nampula city. Nampula city is distributed by 33 neighborhoods and is the third largest city in Mozambique with an estimated population of 746,637 inhabitants (Mozambican National Institute of statistics, projection of 2016).

According to the WHO’s definition, Nampula city is identified as a cholera-endemic area since confirmed cholera cases, resulting from local transmission, have been detected in the last 3 years. Since 2013, Nampula city experienced cholera outbreaks every year. According to the Provincial Epidemiological Department of Nampula, from September 2015 to 10 July 2016 a total of 2,536 cases were recorded in Mozambique and 1,292 of them (50.9%) were recorded in Nampula city. Of them, 854 (66.0%) originated from 6 of the 33 neighborhoods of Nampula city. These neighborhoods are densely populated (around 203,582 people) and are characterized by poor sanitation (i.e open defecation, poor waste collection and degradation of the environment), as well as by difficult access to safe water (i.e irregularities in the supply of potable water to the population and the proliferation of fast food and water selling in inappropriate places without conditions of hygiene and sanitation) [17].

In October 2016, a mass vaccination campaign using two-dose OCVs was organized in the six high-risk neighborhoods of Nampula city. 386,806 OCV doses were needed to target 193,403 individuals. Taking into account population movement in the target area and vaccine wastage, a total of 425,486 doses, considering 10% buffer were requested from the ICG group. The doses of Shanchol™ were transported from the Unicef vaccine supply division in Copenhagen to Nampula via Maputo in the Expanded Program on Immunization (EPI) cool boxes. A mobile door-to-door strategy was used during the two rounds of vaccination. The first round took place between October 3 and 10, 2016, and the second round between October 24 and 31, 2016. All residents of targeted neighborhoods from one year of age and older, including pregnant women, were invited to participate in the mass vaccination campaign. The strategy defined by the Ministry of Health (MoH) was to only give a dose during the second immunization round to those who had been immunized during round one. Vaccination status was assessed by the vaccination team who checked the vaccination cards before administrating the vaccine. For those who didn’t have their vaccination card, control questions were asked to guarantee they had the first dose. During the first round, 209,561 doses of vaccine were administered, and 208,734 during the second round, totaling 418,295 doses. Vaccination teams were composed of at least three persons (one recorder, one mobilizer, and one registrar). The vaccine cold chain was maintained and the vaccines were transported using a sufficient number of vaccine carriers and ice packs.

In addition to the use of OCV to prevent and control cholera in the short-to-medium term, the Mozambican Ministry of Health implemented the following measures: water supply, chlorination of unsafe water sources, and construction of latrines, training of community workers and cleaning of the environment with community involvement.

We conducted a population survey to: estimate vaccination coverage achieved during the OCV campaign; evaluate the frequency of Adverse Events Following Immunization (AEFI) reported during and after the campaign; assess vaccine acceptability; document reasons for non-vaccination; and explored OCV campaign awareness.

## Methods

### Study population and design

The study was conducted in the six high-risk neighborhoods that were targeted during the OCV campaign: Mutauanha, Murrapaniwa, Muatala, Natikiri, Napipine, and Carrupeia. The study population included every person above one year of age who was living in the OCV targeted area at the time of the vaccination campaign.

Survey participants were stratified by age groups: children aged 1–14 years (survey A) and individuals aged 15 years and older (survey B). The households were selected based on housing density using high-resolution optical satellite images obtained from Digital Globe through Google Earth copyright 2016.

To estimate household density from satellite images, we counted the number of households within a sample of 1km x 1km squared in rural areas (100m x 100m in urban areas) within the survey area and then interpolated to estimate housing density in the remainder of the grid. The resulting household density grid was used as a weighting factor to generate the global positioning system (GPS) points. Individual houses were selected on the basis of proximity to each GPS point using Geographic Information Systems software. The list of homes was provided to the survey teams in the form of GPS points to be uploaded to GPS devices. We randomly selected another set of GPS coordinates when the original GPS data corresponded to an empty place or non-residential building. In each household, only one resident was enrolled. The number of individuals in a neighborhood to be interviewed was proportional to the neighborhood’s population size. Based on vaccine coverage results of previous surveys conducted on the African continent [11,13], we estimated that vaccine coverage might approximate 70%. Assuming a 95% significance level and an expected non-response rate of 10%, we calculated that the survey would have a 80% power to estimate the vaccine coverage, with a 6% precision. The required sample size was 600 individuals, including 300 children and 300 adults.

### Data collection

Upon arrival at the household, a list of all members was created, and one individual was randomly selected for interview. After obtaining written consent, data were collected through standardized paper-based questionnaires during face-to-face interviews in *Macua* (local language) or Portuguese depending on the preference of the respondents. As *Macua* is still an oral language; reading and writing *Macua* is not a commonplace occurrence. Thus, the questionnaire were in Portuguese but local interviewers were trained to translate all questions from Portuguese to *Macua* and to conduct the interview in *Macua*.

Pregnancy status was assessed for all women aged 15 to 55 years according to the range of reproductive age for the study area. For children under 12 years, information was given and consent collected from parents or primary caregivers. For those aged between 12–15 years the consent was collected from parents or primary caregivers and the responder could be the child him/herself, but he/she was accompanied by his/her parent or primary caregiver. Cholera immunization status was ascertained through oral reporting (history) and vaccination cards. The questionnaire included sections for socio-demographic status, the number of OCV doses taken, the occurrence of OCV AEFI, reasons for non-vaccination, OCV campaign awareness (informed or not, through what channel) and vaccine acceptability (reasons for taking the vaccine, preferred place for vaccine administration–home, health center). If the selected person was absent, additional visits were scheduled. If the selected person was not present after the third visit, another household was selected.

### Data analysis

Double data entry was done using EpiData version 3.1 (EpiData Association, Denmark) and analyzed with Stata 13.0 (StataCorp., Stata Statistical Software, College Station, TX, USA). OCV coverage for each vaccination round and 95% confidence intervals were calculated and stratified by age groups. Univariate and multivariate analyses of factors associated with vaccine coverage were performed using multinomial logistic regression models. The dependent variable used for the OCV coverage was a categorical variable with three modalities: zero dose (used as reference), one dose, and two doses. All relevant variables with P value <0.20 in the univariate analysis were entered into a multivariate model that was adjusted for gender and age. Multinomial logistic regression was carried out by removing variables one by one in a manual backward procedure using likelihood ratio tests at each step. Variables were kept in the final model if P value<0.05. Age and gender were forced into the final model as adjusting variables. Relative risk ratios (RRR) are presented with their 95% confidence intervals (95% CI). Administrative coverage was defined as the number of doses administered divided by the number of people eligible for that vaccination, based on neighborhood population data from the Provincial Health Directorate (*Direcção Provincial de Saúde*). Individuals were asked about the occurrence of OCV AEFI as well as possible reasons for non-vaccination; we described the distribution using proportion and its 95% confidence interval.

### Ethics statement

The study was approved by the national review board of the Government of Mozambique (Comité Institucional de Bioética para a Saúde do INS) and written informed consent was obtained from all study participants. A parent or guardian of any child participant (under 18 years of age) provided informed consent on their behalf.

## Results

### Characteristics of study population

The survey was conducted from November 2 to 9, 2016. A total of 647 households were visited. In all, 636 (98.29%) residents were enrolled in the survey, 10 (1.54%) refused to participate, and one (0.15%) remained absent after three visits.

Among the 636 survey participants, 298 (46.9%) were children aged between 1- and 14-years-old, whereas 338 (53.1%) were adults aged 15 years and older. The median age of respondents was 17 years (inter-quartile-range (IQR):8–30; range: 1–85 years). All respondents confirmed that they were living in the targeted OCV area at the time of the campaign. The median duration of residency was five years (IQR: 3–10; Range: 0.08–83 years).

The median number of people per household was five (IQR: 4–7; Range: 1–21). For sanitation, 84.6% houses did not share latrines with the neighborhoods. The majority (52.0%) used piped water from their neighbors’ homes. Socio-demographic characteristics are summarized in Table 1.

**Table 1:** Socio-demographic characteristics of respondents, population survey following OCV campaign in the six most vulnerable neighborhoods of Nampula city (*N* = 636), Mozambique, 2016.

Socio-demographic characteristics	n	%
**Gender**		
Female	380	59.9
Male	254	40.1
**Pregnancy (data from female group aged 15 to 55 years)**		
No	193	91.5
Yes	18	8.5
**Age group (years)**		
1–4	82	12.9
5–14	215	33.9
≥15	338	53.2
**Household size (number of persons living in the household)**		
1 to 4	220	34.8
5 to 10	386	61.1
11 and more	26	4.1
**Main occupation of the head of the household**		
Others	172	27.4
Not active/retired	136	21.7
Farmer	78	12.4
Administrative technician	72	11.5
Seller	68	10.8
Medical physician/teacher	43	6.9
Street vendor	31	4.9
Domestic employee	27	4.3
**Education level of the head of the household**		
No education	84	13.5
Primary	206	33.0
Secondary	161	25.8
Superior (academic level)	173	27.7
**Usual source for drinking water**		
Standpipe/Neighbor’s tap	330	52.0
Water from the well	151	23.8
Water piped into the house	134	21.1
Water collected from the river	15	2.4
Bottled water (mineral water)	5	0.8
**Type of toilet/latrine used**		
Latrine without slab	251	39.7
Bucket	205	32.4
Latrine with slab	79	12.5
Open pit	57	9.0
None	35	5.5
Improved latrine	4	0.6
**Place of residence**		
Murrapaniwa	203	31.9
Mutauanha	120	18.9
Muatala	90	14.1
Carrupeia	84	13.2
Napipine	80	12.6
Natikiri	59	9.3

### OCV coverage

Administrative coverage results provided by the MoH were 108.4% and 107.9% after the first and second rounds respectively. Considering the strategy adopted during the campaign which was to vaccinate during the round 2 only people who received their first dose at round 1, the administrative coverage of the second dose would be 99.5%. The lowest administrative estimates were in Carrupeia and Natikiri (see Table 2).

**Table 2:** Administrative coverage (data from the OCV campaign provided by the MoH) in the six most vulnerable neighborhoods of Nampula city, Mozambique, 2016.

Neighborhoods	Target population	Round 1		Round 2	
		n	%	n	%
Murrapaniwa/ Napipine	84,605	90,001	106.4	95,850	113.3
Mutauanha	39,899	46,495	116.5	43,620	109.3
Carrupeia	29,667	27,948	94.2	27,755	93.6
Muatala	27,593	31,779	115.2	30,305	109.8
Natikiri	11,639	13,338	114.6	11,204	96.3

Based on our population survey, the overall vaccination coverage with at least one dose (including card and oral reporting) was 69.5% (95%CI: 51.2–88.2) (Table 3). The full dose coverage (two-dose) was 51.2% (95%CI: 37.9–64.3).

**Table 3:** Vaccination coverage with at least one dose post OCV campaign in the six most vulnerable neighborhoods of Nampula city, Mozambique, 2016.

	Overall (N = 636)			Children (N = 298)			Adults (N = 338)		
	n	%	[95%CI]	n	%	[95%CI]	n	%	[95%CI]
Confirmed via history or vaccination card	451	69.5	[51.2–83.2]	219	69.5	[44.4–86.7]	232	69.5	[51.4–83.1]
Confirmed via the vaccination card only	353	51.5	[36.2–66.4]	179	53.5	[34.2–71.8]	174	49.7	[32.6–66.9]

Overall 77.8% of respondents were able to present a vaccination card. Using only the vaccination card for confirmation of the vaccination status, the two-dose coverage was 40.6% (95%CI: 27.5–55.3).

The highest two-dose coverage was among adult females and the lowest was among adult males (S1 Table).

The proportions of respondents by residence who took at least one dose (including card and oral reporting) were: 78.3% in Murrapaniwa, 70.0% in Mutauanha, 83.3% in Muatala, 41.7% in Carrupeia, 62.5% in Napipine, and 81.4% in Natikiri (S2 Table).

Based on the univariate analysis of the factors associated with the number of OCV doses received, nine independent variables were entered in the complete model: age group (forced), gender (forced), neighborhood, household size, occupation, time to go to health center, transport to health center, water used to drink, type of latrine used. In the final multinomial logistic regression model the proportion of non-vaccinated respondents living in Carrupeia was significantly higher compared with the other neighborhoods and vaccination was higher among households with 5–10 people (Table 4).

**Table 4:** Final multinomial logistic regression model investigating factors associated with the number of OCV doses received, in the six most vulnerable neighborhoods of Nampula city, Mozambique, 2016.

Characteristics	Categories	1 dose versus 0 dose		2 doses versus 0 dose	
		RRR [95% CI]	P-value	RRR [95% CI]	P-value
**Gender**^a^					
	Male	1		1	
	Female	1.0 [0.6–1.7]	0.91	1.4 [0.9–2.1]	0.09
**Age group**^a^ **(years)**					
	1–4	1		1	
	5–14	0.7 [0.3–1.5]	0.40	0.9 [0.5–1.7]	0.74
	≥15	0.5 [0.2–1.0]	0.05	0.7 [0.4–1.4]	0.35
**Neighborhood**					
	Carrupeia	0.6 [0.2–1.4]	0.23	0.2 [0.1–0.4]	<0.001
	Muatala	4.0 [1.7–9.6]	0.002	1.8 [0.9–3.7]	0.10
	Murrapaniwa	2.2 [1.1–4.7]	0.04	1.6 [0.9–2.7]	0.12
	Mutauanha	1		1	
	Napipine	0.6 [0.2–1.6]	0.31	0.8 [0.4–1.4]	0.39
	Natikiri	2.5 [0.9–7.1]	0.09	2.2 [1.0–4.9]	0.06
**Household size (number of persons living in the household)**					
	1 to 4	1		1	
	5 to 10	1.0 [0.6–1.6]	0.93	2.23 [1.47–3.38]	<0.001
	11 and more	0.6 [0.2–2.2]	0.46	1.33 [0.50–3.52]	0.57

^a^ Forced in the model

RRR = Relative Risk Ratio. Note: 9 independent variables were entered in the complete model: age group (forced), gender (forced), neighborhood, household size, occupation, time to go to health center, transport to health center, water used to drink, type of latrine used.

### Reasons for not being vaccinated

Among the non-vaccinated individuals, 83 (44.6%) did not take the vaccine because they were absent when the vaccination team visited the house. In total, 44 (23.6%) mentioned that the vaccination teams did not show up, and 25 (13.4%) indicated that they were not aware about the date or the time of this visit. Among respondents, only one (0.9%) mentioned the vaccine’s bad taste as a reason for not taking the second dose (**Table 5**).

**Table 5:** Reasons for non-vaccination (oral reporting), post OCV campaign survey in the six most vulnerable neighborhoods of Nampula city, Mozambique, 2016.

	n	%
**Resons for non-vaccination**	***N* = 186**	
Absent when the vaccination team came	83	44.6
Vaccination teams did not visit the house	44	23.6
Aware of campaign, but date or time of vaccination team’s visit unknown	25	13.4
Unaware of the vaccination campaign	14	7.5
Had no time	9	4.8
Was not in a good state to take the vaccine	6	3.2
Declared that did not need to be vaccinated	4	2.1
No faith in the vaccine	4	2.1
Unaware she was eligible for the vaccine (pregnancy)	2	1.1
Head of household did not authorize it	1	0.5
Other reasons	5	2.7
**Reasons for non taking the second dose**	***N* = 118**	
Absent when vaccination team came	51	43.2
Vaccination teams did not come back	39	33.0
Unaware cholera vaccination needs two doses	10	8.4
Experienced adverse event with first dose	3	2.5
Bad taste	1	0.8
Had no time	1	0.8
Date or time of vaccination team’s visit unknown	1	0.8
Was not in a good state to take the vaccine	1	0.8

### Frequency of OCV AEFI

OCV adverse events following the first dose (symptoms developed between few hours to 15 days after the administration of the first dose) were reported by 47 (10.4%; 95%CI: 6.9–16.2) vaccinated individuals. No AEFI was reported after taking the second dose.

**Fig 1:**
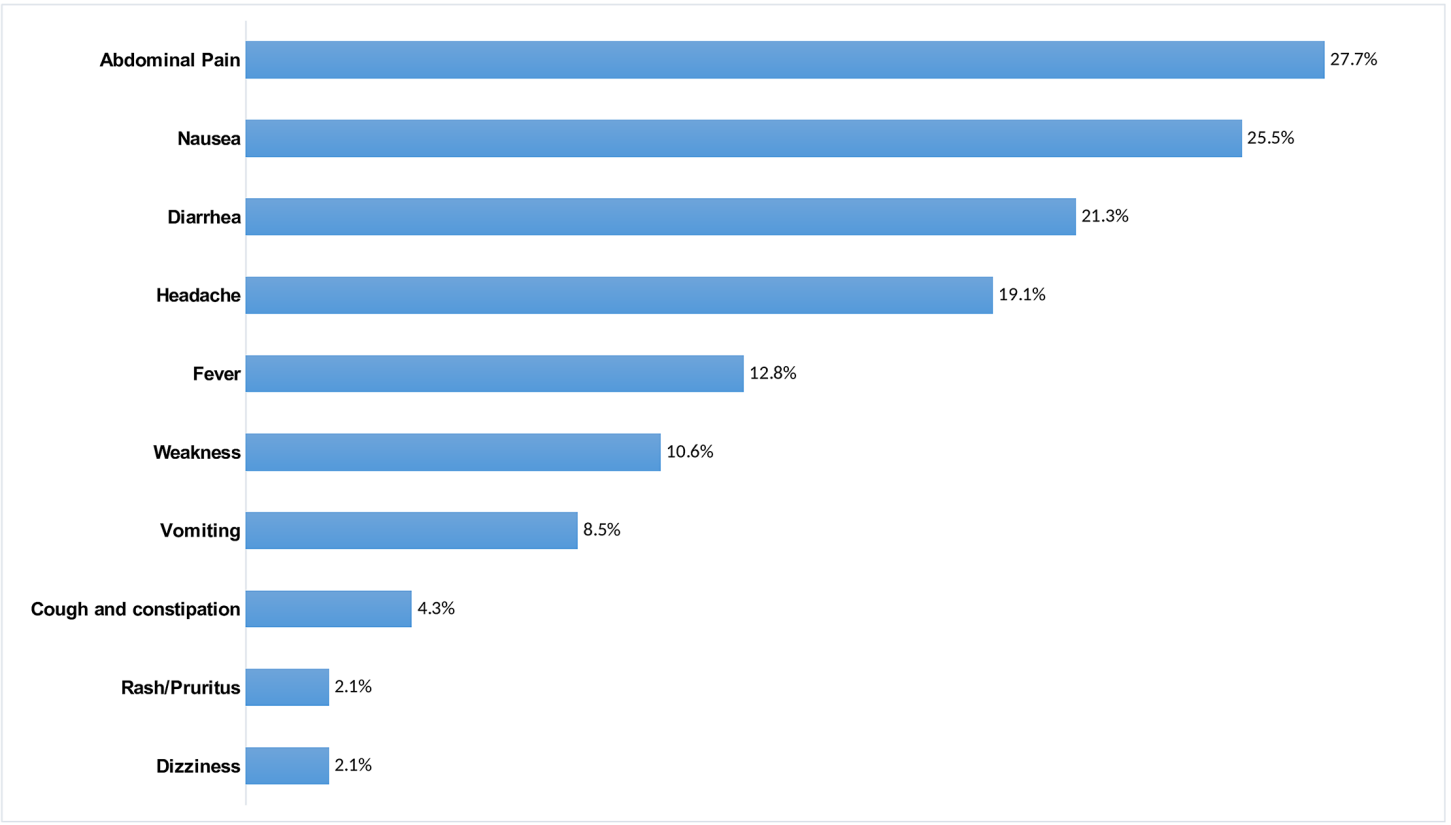
AEFI oral reporting (N = 47), post OCV campaign survey in the six most vulnerable neighborhoods of Nampula city, Mozambique, 2016.

The AEFIs reported were all considered non-serious and most were non-specific, and included abdominal pain, nausea, and diarrhea (Fig 1). Among individuals who reported AEFIs, five (0.6%) were children aged between 1- and 4-years-old, 12 (3.0%) were aged between 5- and 14-years-old, and 30 (7.2%) were adults. No one went to a health facility, considering the clinical symptoms as non-serious.

### OCV campaign awareness and vaccine acceptability

The 451 vaccinated individuals who were interviewed for acceptability, mentioned as main information sources: the neighborhood’s main leader (31.3%), social mobilizers (19.9%), and radio or television (15.5%). Of the 451, 78 (17.3%) mentioned that they did not receive any information before the start of the campaign.

Of the 451 individuals who took at least one dose and were interviewed for vaccine acceptability, 303 (67.5%) stated that they took the vaccine because they considered cholera as a serious disease. The second most common reason was reassurance given by the neighborhood’s main leader that the vaccine was safe (14.9%). While 436 (96.7%) of vaccine recipients felt comfortable receiving the vaccine at home, 80 (17.7%) would rather have been vaccinated in a health facility, and 113 (25.1%) in a community place near their home.

## Discussion

The overall vaccination coverage with at least one dose (including card and oral reporting) was 69.5% (95%CI: 51.2–88.2). Vaccine coverage did not vary according to age group or sex, but did differ by neighborhood. The two-doses coverage was lower in Carrupeia, Muatala and Napipine. These low two-doses coverage especially in Carrupeia, may not be attributed to the community unwillingness to be vaccinated as the majority of respondents were aware about the OCV Campaign. The most frequent reason for non-vaccination or incomplete vaccination was the absence of residents during the vaccination team's visit. The unclear boundary of Carrupeia neighborhood may have been a source of difficulty for the vaccination team to reach all households. Indeed, in urban contexts such as Nampula city, with uncontrolled sprawling settlements and frequent population movements, it may be difficult to have a clear map of the neighborhood and to know the location of the each house, which would be necessary for an exhaustive door-to-door vaccination campaign. To compensate for the absence of clear administrative neighborhood delimitations and lack of available household listing, we developed a sampling method for the coverage survey using GPS devices and worked in close collaboration with the community leaders who provided precious guidance on the topography of the targeted area. They also facilitated community acceptance.

In other similarly challenging African setting, authors have reported a higher vaccine coverage with at least one dose of more than 70% from OCV campaigns, such as from Guinea [11,18], Malawi [19] and from Beira, Mozambique [20]. Nonetheless, during the OCV campaign in Nampula all OCV doses were used and the administrative coverage was higher than 100% in all neighbourhoods except for Carrupeia and Natikiri.

Authors highlighted that insufficient strategic planning and low community awareness are important limiting factors for achieving high vaccination coverage [21].

The OCV campaign implemented in Nampula used a door-to-door strategy, which is the routine strategy of MoH for polio campaigns. This strategy has been designed to decrease the risk of vaccinating individuals from neighborhoods situated outside the targeted area, and to maximize the protection induced by a two-dose regimen. Following the recommendations of the National EPI, the second dose of vaccine could only be administered to those individuals able to present a written documentation or answered control questions confirming that they already received the first dose. When comparing those who took two-doses and those who took zero dose, the results from the multivariate analysis showed that Carrupeia was more affected by this strategy. Other than the neighborhood, the household size appeared to be related to the number of doses (higher two-dose coverage for size 5–10 people compared with 1–4 people). This result need to be clarified. Overall, we can say that the door-to-door strategy has resulted in missing opportunities to deliver at least one OCV dose in some of the targeted neighborhoods.

It is known that indirect effects of OCV play an important role in reducing disease transmission in the community. Recent mathematical models suggest that vaccinating at least 50% of a population exposed to *Vibrio cholerae* could reduce the incidence of cholera disease by up to 88% in the first year following vaccination [22]. Assuming these modelled data are applicable to an actual endemic setting as here in Nampula and that the vaccine was evenly distributed, vaccination coverage with at least one dose should be high enough to protect the targeted population through direct and indirect effects from major outbreaks at least for the next two years. However, two-dose coverage we found is likely too low to confer the full five-year protection shown for the two-dose regimen in randomized clinical trials [23]. The authorities in Mozambique should plan for another campaign in three years’ time to re-establish protection of the community against cholera.

As with previous OCV campaigns [11,20,24] few adverse events were reported. The low number of AEFI reported by the community contributed to good vaccine acceptability [25]. This is corroborated by the high percentage of people declaring that cholera is a serious disease and that protection conferred by the vaccine is important.

Our findings highlight the need to improve community awareness, provide the community with timely information before the start of the campaign, and improve logistics (planners, dates, and venues) to maximize vaccination coverage. As recommended by others [14,21], the use of an alternative approach, such as the combination of delivery strategies (fixed posts and mobile teams), should help to catch individuals harder to reach due to their daily activities. Finally, our findings demonstrated that the implementation of a mass vaccination campaign using a mobile door-to-door strategy remains a viable option in densely-populated urban settings. These findings are encouraging and complement a previous OCV campaign in the coastal city of Beira in 2003 [20,26] where OCV coverage was almost the same. Our experience shows that OCV campaigns can be successfully implemented and well-accepted in non-emergency contexts in order to prevent cholera outbreaks.

## Supporting information

S1 Table Number of OCV doses received (oral reporting and vaccination card) stratified by age group and gender in the six most vulnerable neighborhoods of Nampula city, Mozambique, 2016. (DOCX)

S2 Table Number of OCV doses received (oral reporting and vaccination card) stratified by place of residence in the six most vulnerable neighborhoods of Nampula city, Mozambique, 2016. (DOCX)

S1 File Questionnaire used for the coverage survey in Nampula. (RAR)

## References

[1] Ali M, Lopez AL, You Y, Kim YE, Sah B, Maskery B, et al The global burden of cholera. Bull World Health Organ. 2012;90: 209–218. 10.2471/BLT.11.093427 22461716 PMC3314202

[2] Mengel MA. Cholera in Africa: new momentum in fighting an old problem. Trans R Soc Trop Med Hyg. 2014;108: 391–392. 10.1093/trstmh/tru077 24836060

[3] Msyamboza KP, Kagoli M, M’bang’ombe M, Chipeta S, Masuku HD. Cholera outbreaks in Malawi in 1998–2012: social and cultural challenges in prevention and control. J Infect Dev Ctries. 2014;8: 720–726. 10.3855/jidc.3506 24916870

[4] Langa JP, Sema C, De Deus N, Colombo MM, Taviani E. Epidemic waves of cholera in the last two decades in Mozambique. J Infect Dev Ctries. 2015;9: 635–641. 10.3855/jidc.6943 26142674

[5] Gujral L, Sema C, Rebaudet S, Taibo CLA, Manjate AA, Piarroux R, et al Cholera epidemiology in Mozambique using national surveillance data. J Infect Dis. 2013;208: S107–S114. 10.1093/infdis/jit212 24101638

[6] Verma R, Khanna P, Chawla S. Cholera vaccine: new preventive tool for endemic countries. Hum Vaccines Immunother. 2012;8: 682–684. 10.4161/hv.19083 22634452

[7] Ali M, Debes AK, Luquero FJ, Kim DR, Park JY, Digilio L, et al Potential for Controlling Cholera Using a Ring Vaccination Strategy: Re-analysis of Data from a Cluster-Randomized Clinical Trial. PLoS Med. 2016;13: e1002120 10.1371/journal.pmed.1002120 27622507 PMC5021260

[8] Desai SN, Pezzoli L, Martin S, Costa A, Rodriguez C, Legros D, et al A second affordable oral cholera vaccine: implications for the global vaccine stockpile. Lancet Glob Health. 2016;4: e223–e224. 10.1016/S2214-109X(16)00037-1 27013303

[9] Desai SN, Pezzoli L, Alberti KP, Martin S, Costa A, Perea W, et al Achievements and challenges for the use of killed oral cholera vaccines in the global stockpile era. Hum Vaccines Immunother. 2016; 00–00. 10.1080/21645515.2016.1245250 PMC5360144 27813703

[10] WHO. Weekly epidemiological record—Cholera vaccines: WHO position paper–August 2017 [Internet]. Geneva; 2017 Aug pp. 477–500. Report No.: 92. Available: http://apps.who.int/iris/bitstream/10665/258763/1/WER9234.pdf?ua=1

[11] Luquero FJ, Grout L, Ciglenecki I, Sakoba K, Traore B, Heile M, et al First outbreak response using an oral cholera vaccine in Africa: vaccine coverage, acceptability and surveillance of adverse events, Guinea, 2012. PLoS Negl Trop Dis. 2013;7: e2465 10.1371/journal.pntd.0002465 24147164 PMC3798604

[12] Ivers LC, Teng JE, Lascher J, Raymond M, Weigel J, Victor N, et al Use of oral cholera vaccine in Haiti: a rural demonstration project. Am J Trop Med Hyg. 2013;89: 617–624. 10.4269/ajtmh.13-0183 24106187 PMC3795090

[13] Abubakar A, Azman AS, Rumunu J, Ciglenecki I, Helderman T, West H, et al The first use of the global oral cholera vaccine emergency stockpile: lessons from South Sudan. PLoS Med. 2015;12: e1001901 10.1371/journal.pmed.1001901 26576044 PMC4648513

[14] Lam E, Al-Tamimi W, Russell SP, Butt MO-UI, Blanton C, Musani AS, et al Oral Cholera Vaccine Coverage during an Outbreak and Humanitarian Crisis, Iraq, 2015. Emerg Infect Dis. 2017;23: 38–45. 10.3201/eid2301.160881 27983502 PMC5176248

[15] Ngwa MC, Liang S, Kracalik IT, Morris L, Blackburn JK, Mbam LM, et al Cholera in Cameroon, 2000–2012: Spatial and Temporal Analysis at the Operational (Health District) and Sub Climate Levels. PLoS Negl Trop Dis. 2016;10: e0005105 10.1371/journal.pntd.0005105 27855171 PMC5113893

[16] Msyamboza KP, M’bang’ombe M, Hausi H, Chijuwa A, Nkukumila V, Kubwalo HW, et al Feasibility and acceptability of oral cholera vaccine mass vaccination campaign in response to an outbreak and floods in Malawi. Pan Afr Med J. 2016;23: 203 doi: 10.11604/pamj.2016.23.203.8346 27347292 10.11604/pamj.2016.23.203.8346 PMC4907756

[17] Admiraal R, Doepel D. Using baseline surveys to inform interventions and follow-up surveys: a case-study using the Nampula Province Water, Sanitation, and Hygiene Program. J Water Sanit Hyg Dev. 2014;4: 410–421.

[18] Ciglenecki I, Sakoba K, Luquero FJ, Heile M, Itama C, Mengel M, et al Feasibility of mass vaccination campaign with oral cholera vaccines in response to an outbreak in Guinea. PLoS Med. 2013;10: e1001512 10.1371/journal.pmed.1001512 24058301 PMC3769208

[19] Sauvageot D, Saussier C, Gobeze A, Chipeta S, Mhango I, Kawalazira G, et al Oral cholera vaccine coverage in hard-to-reach fishermen communities after two mass Campaigns, Malawi, 2016. Vaccine. 2017;35: 5194–5200. 10.1016/j.vaccine.2017.07.104 28803712 PMC5594244

[20] Cavailler P, Lucas M, Perroud V, McChesney M, Ampuero S, Guérin PJ, et al Feasibility of a mass vaccination campaign using a two-dose oral cholera vaccine in an urban cholera-endemic setting in Mozambique. Vaccine. 2006;24: 4890–4895. 10.1016/j.vaccine.2005.10.006 16298025

[21] Schaetti C, Ali SM, Chaignat C-L, Khatib AM, Hutubessy R, Weiss MG. Improving community coverage of oral cholera mass vaccination campaigns: lessons learned in Zanzibar. PloS One. 2012;7: e41527 10.1371/journal.pone.0041527 22844489 PMC3402403

[22] Dimitrov DT, Troeger C, Halloran ME, Longini IM, Chao DL. Comparative effectiveness of different strategies of oral cholera vaccination in bangladesh: a modeling study. PLoS Negl Trop Dis. 2014;8: e3343 10.1371/journal.pntd.0003343 25473851 PMC4256212

[23] Bhattacharya SK, Sur D, Ali M, Kanungo S, You YA, Manna B, et al 5 year efficacy of a bivalent killed whole-cell oral cholera vaccine in Kolkata, India: a cluster-randomised, double-blind, placebo-controlled trial. Lancet Infect Dis. 2013;13: 1050–1056. 10.1016/S1473-3099(13)70273-1 24140390

[24] Phares CR, Date K, Travers P, Déglise C, Wongjindanon N, Ortega L, et al Mass vaccination with a two-dose oral cholera vaccine in a long-standing refugee camp, Thailand. Vaccine. 2016;34: 128–133. 10.1016/j.vaccine.2015.10.112 26549363

[25] Rafael F, Chicumbe S, Cavailler P, Barata A, Langa JPM. Passive, health center-based assessment of adverse events following oral cholera immunization in Nampula city, Mozambique. Vaccine. 2017; 10.1016/j.vaccine.2017.07.033 28899627

[26] Lucas ME, Deen JL, von Seidlein L, Wang X-Y, Ampuero J, Puri M, et al Effectiveness of mass oral cholera vaccination in Beira, Mozambique. N Engl J Med. 2005;352: 757–767. 10.1056/NEJMoa043323 15728808

