# Supplementary material for: Oral cholera vaccine coverage during a preventive door-to-door mass vaccination campaign in Nampula, Mozambique
Source: PLoS One. 2018 Oct 3;13(10):e0198592. doi: 10.1371/journal.pone.0198592 (PMC6169854; doi:10.1371/journal.pone.0198592)
Supplement: S1 Table — (DOCX) [file pone.0198592.s001.docx]

**S1** **Table. Number of OCV doses received (oral reporting and vaccination card) stratified by age group and gender in the six most vulnerable neighborhoods of Nampula city, Mozambique, 2016**

| **Characteristics** | **Overall**  **(N = 636)** | | |  | **Children**  **(N = 298)** | | | |  | | **Adults**  **(N = 338)** | | | |  |
| --- | --- | --- | --- | --- | --- | --- | --- | --- | --- | --- | --- | --- | --- | --- | --- |
|  | **n** | **%** | **[95% CI]** |  | **n** | **%** | **[95% CI]** |  | | **n** | | **%** | **[95%CI]** |  |  |
| **All respondents** |  |  |  |  |  |  |  |  | |  | |  |  |  |  |
| 2 OCV doses taken | 332 | 51.2 | [37.9– 64.3] |  | 157 | 49.4 | [31.9 – 67.1] |  | | 175 | | 52.7 | [37.2 – 67.7] |  |  |
| 1 OCV dose taken | 118 | 18.1 | [11.5– 27.4] |  | 62 | 20.1 | [14.2 – 27.7] |  | | 56 | | 16.4 | [9.2 – 27.7] |  |  |
| 0 OCV dose taken | 186 | 30.7 | [17.1– 48.7] |  | 79 | 30.5 | [13.3 – 55.6] |  | | 107 | | 30.8 | [17.3 – 48.8] |  |  |
| **Female respondents** |  |  |  |  |  |  |  |  | |  | |  |  |  |  |
| 2 OCV doses taken | 208 | 53.0 | [39.0– 66.6] |  | 88 | 48.9 | [32.0– 66.1] |  | | 120 | | 56.2 | [13.4– 50.3] |  |  |
| 1 OCV dose taken | 66 | 17.0 | [11.0– 25.3] |  | 33 | 18.9 | [13.3– 26.3] |  | | 33 | | 15.4 | [8.4– 26.6] |  |  |
| 0 OCV dose taken | 106 | 30.0 | [15.3– 50.4] |  | 46 | 32.1 | [13.6– 58.6] |  | | 60 | | 28.3 | [13.4– 50.3] |  |  |
| **Male respondents** |  |  |  |  |  |  |  |  | |  | |  |  |  | |
| 2 OCV doses taken | 122 | 48.1 | [32.5– 64.1] |  | 67 | 49.7 | [30.3– 69.2] |  | | 55 | | 46.5 | [28.4– 65.6] |  |  |
| 1 OCV dose taken | 52 | 20.1 | [10.9– 33.9] |  | 29 | 21.7 | [11.7– 37.1] |  | | 23 | | 18.2 | [9.3– 32.7] |  |  |
| 0 OCV dose taken | 80 | 31.8 | [16.4– 52.6] |  | 33 | 28.4 | [10.9– 56.2] |  | | 47 | | 35.3 | [18.1– 57.3] |  |  |
