# Supplementary material for: Oral cholera vaccine coverage during a preventive door-to-door mass vaccination campaign in Nampula, Mozambique
Source: PLoS One. 2018 Oct 3;13(10):e0198592. doi: 10.1371/journal.pone.0198592 (PMC6169854; doi:10.1371/journal.pone.0198592)
Supplement: S2 Table — (DOCX) [file pone.0198592.s002.docx]

**S2 Table. Number of OCV doses received (oral reporting and vaccination card) stratified by place of residence in the six most vulnerable neighborhoods of Nampula city, Mozambique, 2016**

| **Neighborhoods** | **n** | **%** |  |
| --- | --- | --- | --- |
| **Murrapaniwa** |  |  |  |
| 2 OCV doses taken | 116 | 57.1 |  |
| 1 OCV dose taken | 43 | 21.2 |  |
| 0 OCV dose taken | 44 | 21.7 |  |
| **Mutauanha** |  |  |  |
| 2 OCV doses taken | 69 | 57.5 |  |
| 1 OCV dose taken | 15 | 12.5 |  |
| 0 OCV dose taken | 36 | 30.0 |  |
| **Muatala** |  |  |  |
| 2 OCV doses taken | 46 | 51.1 |  |
| 1 OCV dose taken | 28 | 31.1 |  |
| 0 OCV dose taken | 16 | 17.8 |  |
| **Carrupeia** |  |  |  |
| 2 OCV doses taken | 22 | 26.2 |  |
| 1 OCV dose taken | 13 | 15.5 |  |
| 0 OCV dose taken | 49 | 58.3 |  |
| **Natikiri** |  |  |  |
| 2 OCV doses taken | 37 | 62.7 |  |
| 1 OCV dose taken | 11 | 18.6 |  |
| 0 OCV dose taken | 11 | 18.6 |  |
| **Napipine** |  |  |  |
| 2 OCV doses taken | 42 | 52.5 |  |
| 1 OCV dose taken | 8 | 10.0 |  |
| 0 OCV dose taken | 30 | 37.5 |  |
